# Dementia-related genetic variants in an Italian population of early-onset Alzheimer’s disease

**DOI:** 10.3389/fnagi.2022.969817

**Published:** 2022-09-05

**Authors:** Anna Bartoletti-Stella, Martina Tarozzi, Giacomo Mengozzi, Francesca Asirelli, Laura Brancaleoni, Nicola Mometto, Michelangelo Stanzani-Maserati, Simone Baiardi, Simona Linarello, Marco Spallazzi, Roberta Pantieri, Elisa Ferriani, Paolo Caffarra, Rocco Liguori, Piero Parchi, Sabina Capellari

**Affiliations:** ^1^Department of Experimental Diagnostic and Specialty Medicine (DIMES), University of Bologna, Bologna, Italy; ^2^IRCCS Istituto delle Scienze Neurologiche di Bologna, Bellaria Hospital, Bologna, Italy; ^3^Department of Medical Science and Surgery (DIMEC), University of Bologna, Bologna, Italy; ^4^Neurologia e Rete Stroke Metropolitana, Ospedale Maggiore, Bologna, Italy; ^5^UOC Neurologia, Ospedale Guglielmo da Saliceto, Piacenza, Italy; ^6^Programma Cure Intermedie - Azienda USL di Bologna, Bologna, Italy; ^7^U.O. di Neurologia, Azienda Ospedaliero-Universitaria, Parma, Italy; ^8^UOC Psicologia Clinica Ospedaliera, Ospedale Bellaria, Azienda USL di Bologna, Bologna, Italy; ^9^Unità di Neuroscienze, Università di Parma, Parma, Italy; ^10^Department of Biomedical and NeuroMotor Sciences (DIBINEM), University of Bologna, Bologna, Italy

**Keywords:** Alzheimer’s disease, early onset Alzheimer disease, next generation sequencing, genetic heterogeneity, mutation screening

## Abstract

Early-onset Alzheimer’s disease (EOAD) is the most common form of early-onset dementia. Although three major genes have been identified as causative, the genetic contribution to the disease remains unsolved in many patients. Recent studies have identified pathogenic variants in genes representing a risk factor for developing Alzheimer’s disease (AD) and in causative genes for other degenerative dementias as responsible for EOAD. To study them further, we investigated a panel of candidate genes in 102 Italian EOAD patients, 45.10% of whom had a positive family history and 21.74% with a strong family history of dementia. We found that 10.78% of patients carried pathogenic or likely pathogenic variants, including a novel variant, in *PSEN1*, *PSEN2*, or *APP*, and 7.84% showed homozygosity for the ε4 *APOE* allele. Additionally, 7.84% of patients had a moderate risk allele in *PSEN1*, *PSEN2*, or *TREM2* genes. Besides, we observed that 12.75% of our patients carried only a variant in genes associated with other neurodegenerative diseases. The combination of these variants contributes to explain 46% of cases with a definite familiarity and 32% of sporadic forms. Our results confirm the importance of extensive genetic screening in EOAD for clinical purposes, to select patients for future treatments and to contribute to the definition of overlapping pathogenic mechanisms between AD and other forms of dementia.

## Introduction

Alzheimer’s disease (AD) is the most common form of dementia in the elderly and is associated with environmental and genetic components. Approximately 10% of patients with AD have an early onset disease (<65 years, EOAD) (Cacace et al., 2016) in which the heritability is between 92 and 100% (Wingo et al., 2012). Conversely, late onset AD (LOAD) is genetically more complex with heritability estimates of 58–70% (Gatz et al., 2006; Wingo et al., 2012). Still, the genetic factors identified account only for a portion of the genetic basis of the disease. To date, only about 33% of the genetic variance in sporadic AD is accounted for by common variants, and ultra-rare, rare and low-frequency variants that typically have a more harmful impact on protein function may be significant to the ‘missing heritability’ of AD (Khani et al., 2022). A proportion of 35–60% of EOAD patients have at least one affected first-degree relative (Campion et al., 1999; Brickell et al., 2006), while in 10–15%, the inheritance is autosomal dominant. Pathogenic variants in presenilin 1 (*PSEN1*), presenilin 2 (*PSEN2*) and amyloid precursor protein (*APP*) genes, the main autosomal dominant genetic causes, explain 5–10% of EOAD and about 50% of familial forms (Cacace et al., 2016). New data suggest that a mix of common and rare variants may cause unexplained cases that follow a non-Mendelian pattern of inheritance (Mrdjen et al., 2019). On the other hand, sporadic LOAD is considered a complex trait for which approximately 40 disease-associated genes/loci have been reported, exerting moderate to high pathogenic effects (Kunkle et al., 2019; Bellenguez et al., 2020), which have often been confirmed in EOAD (Cochran et al., 2019; Lacour et al., 2019). Among these, the ε4 allele of the apolipoprotein E (*APOE*) gene is not only a major genetic risk factor for LOAD, increasing the risk of disease by 3-fold in heterozygous and 15-fold in homozygous carriers (Genin et al., 2011), but also for EOAD. In these patients, the risk of disease increases more significantly than in LOADs in both ε4 homozygous and heterozygous carriers with a positive family history (Cochran et al., 2019). Furthermore, truncating and pathogenic variants in the ATP binding cassette subfamily A member 7 (*ABCA7*), Sortilin-related receptor 1, (*SORL1*), and Triggering receptor expressed on myeloid cells 2, (*TREM2*) genes were shown to act in a Mendelian mode in EOAD (Bellenguez et al., 2017). Additionally, a role in the disease was demonstrated for variants in genes involved in other types of neurodegenerative dementia (Cacace et al., 2016; Bartoletti-Stella et al., 2018, 2020; Sassi et al., 2018; Bonvicini et al., 2019; Giau et al., 2019; Wang et al., 2019; Park et al., 2020; Tarozzi et al., 2022), supporting the hypothesis of overlapping molecular mechanisms and a shared genetic basis. It is also likely that variants specific to individual populations, and thus difficult to detect with genome-wide ass1ociation study (GWAS) approaches unless the population is homogenous, may explain at least a portion of the missing heritability of EOAD. In addition, other inheritance patterns should be considered, e.g., autosomal recessive loci might cause EOAD (Moreno-Grau et al., 2021).

To investigate the role of rare variants in a well-characterized EOAD population of Italian origin, we analyzed 102 EOAD patients by a Next-Generation Sequencing (NGS) multigene panel covering causal and risk factor genes for AD and genes related to other forms of dementia.

## Materials and methods

### Participants

The EOAD consecutive unrelated patients referred to the Cognitive Disorders and Dementia Center of the UOC Clinica Neurologica, IRCCS Institute of Neurological Sciences of Bologna, from 2004 to 2019, either as outpatients, inpatients, or sent for genetic analysis, were recruited.

The AD was diagnosed according to the 2011 NIA-AA and International Working Group 2 criteria (McKhann et al., 2011; Dubois et al., 2014). All patients had evidence of AD pathophysiological process as defined by the presence of a characteristic AD CSF biomarker profile, calculated using in-house cutoff values [phosphorylated (p)-tau/Aβ42 ratio > 0.108 and total Aβ42/Aβ40 ratio < 0.68] as reported in Abu-Rumeileh et al. (2018). The strength of a patient’s family history was quantified with the modified Goldman score (Goldman et al., 2005) as reported in Cochran et al. (2019). Briefly, S1 Score: at least three people in two generations affected by EOAD, with one being a first-degree relative of the other two; S1.5 is the same as S1 but LOAD instead of EOAD; S2: at least three relatives with AD without complete autosomal dominant inheritance; S3: a single first- or second-degree family member affected with EOAD; S3.5 same as S3 but LOAD instead of EOAD. We considered patients with strong familiarity those with S1 and S1.5 scores, and with moderate family history if linked to S2, S3 or S3.5 scores.

### Next generation sequencing

Genomic DNA from peripheral blood was isolated using the Maxwell 16 extractor (Promega, Madison, WI, United States) and quantified using the Quantus Fluorometer (Promega) with QuantiFluor double-stranded DNA system. Genetic screening was performed by Next Generation Sequencing (NGS) multigene panels, by using either one of the following panels: amplicon-based Illumina panel (Bartoletti-Stella et al., 2018) and probe-based Illumina panel (Truseq Neurodegeneration Illumina). Sequencing was performed on a MiSeq or NextSeq 500 sequencer using Illumina V2 reagent kit, with 2 × 150 bp paired end read cycles. Sequencing data were analyzed with an in-house bioinformatic pipeline: trimming and quality assessment of raw reads was performed with Trimmomatic (Bolger et al., 2014), mapping was performed with Burrows-Wheeler Aligner (Li and Durbin, 2009) using bwa-mem algorithm on the reference genome GRCh37/Hg19. Variant calling was performed with Strelka2 (Kim et al., 2018). Variant filtration and depth of coverage analysis were performed using Genome Analysis Toolkit (GATK) v4 (McKenna et al., 2010).

### Variant classification

Variants annotation and selection were performed with BaseSpace Variant Interpreter (Illumina, CA, United States). Variants [single-nucleotide variants (SNV) and small indels] in the coding region or in the flanking 7 bp were filtered and selected with the following criteria: (i) sequence read depth at least 10× (ii) for heterozygous variants, an allelic balance value in the range of 0.30 and 0.70 (iii) Minor Allele Frequency (MAF) in the European population reported on the Genome Aggregation Database (GnomAD) (Karczewski et al., 2020) < 1%. Selected variants were classified according to the American College of Medical Genetics and Genomics guidance for the interpretation of sequence variants (Richards et al., 2015). Those reported in ClinVar (Landrum et al., 2018) or HGMD (Stenson et al., 2003) databases were classified accordingly as known disease-causing variants (Pathogenic) or variants of uncertain significance (VUS). To predict the pathogenicity of never reported variants, we performed several *in silico* analyses. The functional consequences of missense variants were predicted by four *in silico* models: Polyphen2 (Adzhubei et al., 2013), M-CAP (Jagadeesh et al., 2016), CADD v1.4 (Kircher et al., 2014), and MutationTaster (Schwarz et al., 2014), intronic splicing variants by: NetGene2 (Hebsgaard et al., 1996), MaxEntScan and Human Splicing Finder^1^, while silent variants by MutationTaster (Schwarz et al., 2014), CADD (Kircher et al., 2014), and FATHMM XF^2^. Allele frequencies were compared with those reported in the Genome Aggregation Database (GnomAD v2.1.1). Variant calling files (VCF) related to the analyzed genomic regions were reported in the Supplementary File.

### Copy number variation analysis in Alzheimer’s disease causative genes

A preliminary *in silico* copy number variation analysis was performed on the sequencing data using the CNVkit (Talevich et al., 2016). Results for *APP*, *PSEN1*, and *PSEN2* genes were validated using a Multiplex Ligation-dependent Probe Amplification (MLPA) assay (MRC Holland). The results of the MLPA analysis were analyzed with Coffalyzer.net.

### *APOE* genotyping

Genotyping of *APOE* was performed by restriction fragment length polymorphism according to Wenham et al. (1991).

### Clinical classification of variants

According to Cochran et al. (2019), pathogenic and likely pathogenic variants were returned as “diagnostic.” Variants classified in ClinVar or HGMD database as of uncertain significance (VUS) identified in AD-Causative genes or AD-risk factor genes (*TREM2*, *ABCA7*, and *SORL1*) were considered as risk factor alleles. We considered contributor of disease variants reported in ClinVar or HGMD as uncertain significance if found in fEOAD and if enriched in our AD cohort than GnomAD European non-finish population (v.2.1.1), assessed with Fisher’s exact Test and Benjamini–Hochberg false discovery rate correction, *p*-value < 0.05.

### Statistical analysis

Variant zygosity was extracted from the VCF files and allele frequencies of our dataset were compared with those reported in GnomAD. Statistical significance (*p* < 0.05) of variant allele frequencies between our dataset and those reported in GnomAD for the European (non-Finnish) population was assessed with Fisher’s exact Test and Benjamini–Hochberg false discovery rate correction. All tables report only adjusted *p*-values.

## Results

### Study population

The cohort included 102 patients diagnosed with EOAD; in one the diagnosis was neuropathologically confirmed (0.98%), in 72 was defined as “probable” (70.59%), and in 29 as “possible” (28.43%) with evidence of the AD pathophysiological process (McKhann et al., 2011). According to the International Working Group 2 criteria, a “typical” AD phenotype was found in 58 individuals (56.8%), the frontal variant in 15 (14.7%), the logopenic variant in 14 (13.7%), and the posterior variant in 15 (14.7%). The mean of age at onset (AAO) was 56.88 ± 5.84 years, while 10 patients presented the first symptoms before the age of 51. Forty-nine patients were male (48.04%), 46 showed a positive family history (45.10%), of whom 10 were classified as having a strong family history (21.74% of all family cases) (Table 1). Dimensionality reduction plots performed with Principal Component Analysis (PCA) and t-distributed stochastic neighbor embedding (t-SNE, Jaccard similarity used as metric, right panel) show an overall homogenous genetic background in our EOAD cohort, with no confounders caused by the geographical origin of the patients (Supplementary Figure 1).

**TABLE 1:** Clinical features of study population.

Patients/Clinical characteristics	*N* (102)	%
***Gender***		
Male	49	48.04
Female	53	51.96
***Age at onset (y)***		
Mean ± *SD*	56.88 ± 5.84	
***Diagnosis* ^1^**		
Possible	29	28.43
Probable	72	70.59
Certain	1	0.98
***Family history***		
fEOAD	46	45.10
Strong fh (GS 1 or 1,5)	10	21.74
Moderate fh (GS 2-3-3.5)	36	78.26
sEOAD	56	54.90

EOAD, early onset Alzheimer disease; fEOAD, familial EOAD; fh, positive family history, N, number; GS, Goldman score, standard deviation, sEOAD, sporadic EOAD, y, years.

^1^According to the International Working Group 2 criteria (Dubois et al., 2014).

### Analysis of causative genes: *APP, PSEN1*, and *PSEN2*

We found 17 different rare variants (MAF < 0.01) in the AD causative genes *APP*, *PSEN1*, and *PSEN2* (Table 2). Nine (8.82%) patients carried a “diagnostic” variant; seven had a positive family history, although a strong familiarity was established only in two. All identified variants had been previously reported, with the only exception of p.Leu85Phe in the *PSEN1* gene (Table 2), which was not even present in the GnomAD. The carrier had a posterior variant of AD, with positive familial history. Two patients (AD#101 and AD#043) carried two variants, one in the *APP* and *PSEN1* genes, and the second in the *PSEN1* and *PSEN2* genes. No CNV were identified in AD-causative gene. Six rare variants were previously reported in the ClinVar or HGMD database as likely benign or of uncertain significance (*APP* p.Phe435=, *PSEN1* p.Arg35Gln, *PSEN2* p.Arg62His, p.Arg71Trp, p.Met174Val, p.Ser236=) (Table 2).

**TABLE 2:** Rare variants in AD-causative genes identified in this study.

Gene	ID patient	FH score	Nucleotide change	Protein change	Pathogenicity ClinVar/HGMD	Clinical significance	Frequency GnomAD (EU)^1^	*P*-value^2^
*APP*	AD#089	3.5	c.1305C > T	p.Phe435=	Benign/NR	Benign	132/128888	0.23
	AD#101	3.5	c.2137G > A	p.Ala713Thr	Conflicting interpretations of pathogenicity: likely pathogenic (1); uncertain significance (2)/Alzheimer disease	Diagnostic	4/129100	0.01
	AD#010	3.5	c.2229C > T	p.Thr743=	NR/NR/prediction: likely benign^3^	Benign	NR	NA
*PSEN1*	AD#043	0	c.104G > A	p.Arg35Gln	Conflicting interpretation of pathogenicity uncertain significance (3); Benign (1); likely benign (1)/Alzheimer disease?	Risk factor	37/129122	0.08
	AD#102	3.5	c.253C > T	p.Leu85Phe	NR/NR/prediction: probable pathogenic	Diagnostic	NR	NA
	AD#055	1.5	c.275G > C	p.Cys92Ser	Pathogenic/Alzheimer disease	Diagnostic	NR	NA
	AD#001	3.5	c.497T > A	p.Leu166His	NR/Alzheimer disease, early-onset	Diagnostic	NR	NA
	AD#057	3.5	c.617G > C	p.Gly206Ala	Pathogenic/Alzheimer disease	Diagnostic	NR	NA
	AD#012	1	c.791C > T	p.Pro264Leu	Pathogenic/Alzheimer disease	Diagnostic	NR	NA
	AD#002	3.5	c.1172T > C	p.Val391Ala	NR/Alzheimer disease	Diagnostic	NR	NA
	AD#101	3.5	c.1315A > G	p.Ile439Val	NR/Alzheimer disease	Diagnostic	NR	NA
	AD#022	0	c.185G > A	p.Arg62His	Benign/Alzheimer disease?	Benign	300/128852	0.41
*PSEN2*	AD#097	0	c.211C > T	p.Arg71Trp	Benign/Alzheimer disease?	Risk factor	506/129030	0.51
	AD#098	0						
	AD#077	0						
	AD#035	3	c.520A > G	p.Met174Val	Benign/Alzheimer disease?	Contributor of disease	44/129182	<0.0001
	AD#091	3.5						
	AD#043	0	c.668G > C	p.Gly223Ala	NR/Alzheimer disease	Diagnostic	NR	NA
	AD#065	3.5	c.708T > C	p.Ser236=	Benign/NR	Likely benign	791/129088	0.4
	AD#053	0	c.1186C > T	p.Leu396Phe	NR/Alzheimer disease	Diagnostic	1/113608	0.01

AD, Alzheimer disease, FH, family history, NR, Not reported, NA, not applicable.

^1^Population allele frequencies referred to the European (non-Finnish) population reported on GnomAD v2.1.1, expressed as Allele count (Alt/total).

^2^*P*-value Fisher’s exact test, BH correction.

^3^Never reported variants in the *APP* gene not located in the exon 16 and 17 (Cacace et al., 2016) have been considered likely benign. Novel variants have been classified as “likely pathogenic” if at least three tools out of the four used showed potentially pathogenic effects (Supplementary Table 2).

### *APOE* genotype

Eight patients (7.84%), all negative for variants in AD causative genes, carried the *APOE* ε4/ε4 genotype (Cochran et al., 2019). Seven of them had a positive family history, while familiarity was strong in two. Thirty-two (31.37%) patients showed heterozygosity for the *APOE* ε4 allele: thirty carried the *APOE* ε3/ε4 and two the *APOE* ε2/ε4 genotype. The frequency of heterozygosity for the *APOE* ε4 allele was not increased in patients with a positive family history.

### Variants in *TREM2* gene

Like *APOE* ε4, pathogenic variants in *TREM2* also increase a person’s odds of developing late-onset AD from three- to 12-fold (Wolfe et al., 2019).

We identified five different variants in this gene, all singletons, in four patients (Table 3). One patient (AD#026) carried two variants. The variants discovered were not strictly deleterious: four were previously reported in the ClinVar database as benign, and one as VUS.

**TABLE 3:** Rare variants in AD-risk gene *TREM2* identified in this study.

ID patient	FH score	Nucleotide change	Protein change	Pathogenicity ClinVar/HGMD	Clinical classification	Frequency GnomAD (EU)^1^	*P*-value^2^
AD#085	0	c.140G > A	p.Arg47His	Likely benign/Alzheimer disease, increased risk	Risk factor	315/127748	0.18
AD#026	3	c.287C > A	p.Thr96Lys	Benign/frontotemporal dementia, increased risk	Risk factor	130/129182	0.08
		c.632T > C	p.Leu211Pro	Benign/Alzheimer disease, increased risk	Risk factor	144/129164	0.11
AD#045	0	c.407G > A	p.Arg136Gln	Uncertain significance/Alzheimer disease?	Risk factor	17/128820	0.02
AD#089	3.5	c.668C > T	p.Thr223Ile	Benign/Alzheimer disease?	Risk factor	49/129176	0.05

AD, Alzheimer disease, FH, family history. ^1^Population allele frequencies referred to the European (non-Finnish) population reported on GnomAD v2.1.1, expressed as Allele count (Alt/total). ^2^*P*-value Fisher’s exact test, BH correction.

### Variants in dementia-associated genes

Given the common mechanisms previously reported between AD and other neurodegenerative diseases (Bartoletti-Stella et al., 2018; Park et al., 2020), we also collected data on likely pathogenic rare variants in genes that play a role in other dementias.

After applying the filtering criteria, we identified 33 different rare variants in 12 genes (Table 4). Variants previously reported as pathogenic or likely pathogenic were identified in patients with motor neuron disease (*FUS* p.Gly144_Tyr149del, *DCTN1* p.Arg997Trp, *OPTN* p. Gln314Leu and c.1401 + 4A > G, *SQSTM1* p.Pro392Leu) and amyotrophic lateral sclerosis (ALS)-Charcot-Marie-Tooth disease type 4 (*FIG4* p.Gln823Ter). Five previously unreported variants were classified as likely pathogenic (Table 5) by *in silico* prediction. These variants map to the *CCNF*, *DCTN1*, and *NOTCH3* genes, which are involved in the ALS/frontotemporal dementia (FTD) spectrum (Supplementary Table 2). In addition, *NOTCH3* variants have been previously associated with AD risk (Patel et al., 2019).

**TABLE 4:** Rare variants in other dementia causative genes identified in this study.

Gene	ID patient	FH score	Nucleotide change	Protein change	Pathogenicity ClinVar/HGMD	Frequency GnomAD (EU)^1^	*P*-value^2^
*CCNF*	AD#020	0	c.353T > C	p.Val118Ala	NR/NR/prediction – Likely pathogenic	1/111550	0.02
	AD#079	1.5	c.656T > C	p.Leu219Pro	NR/NR/prediction – Likely pathogenic	5/111832	0.03
*CHCHD10*	AD#068	0	c.354C > A	p.Asp118Glu	Uncertain significance/NR	NR	NA
*CSF1R*	AD#081	0	c.1400C > T	p.Thr467Met	NR/NR/prediction – Likely benign	5/113734	0.03
	AD#057	3.5	c.1477A > G	p.Ser493Gly	NR/NR/prediction – Likely benign	NR	NA
	AD#044	0	c.2850C > A	p.His950Gln	NR/NR/prediction – Likely benign	2/113066	0.02
			c.2851C > A	p.Leu951Met	NR/NR/prediction – Likely benign	2/113102	0.02
*DCTN1*	AD#102	3.5	c.586A > G	p.Ile196Val	Conflicting interpretations of pathogenicity. Uncertain significance (2); Benign (6)/abnormal cellular organization.	649/105456	1
	AD#030	0	c.1361T > C	p.Val454Ala	NR/NR/prediction – Likely pathogenic	NR	NA
	AD#100	3	c.1480G > A	p.Ala494Thr	Uncertain significance/amyotrophic lateral sclerosis, phenotype modifiers?	4/128890	0.03
	AD#067	0	c.1555A > G	p.Lys519Glu	NR/NR prediction: Likely pathogenic	NR	NA
	AD#070	1	c.2278A > G	p.Met760Val	Conflicting interpretation of pathogenicity Uncertain significance (2) Benign (2) Likely benign (1)/NR	10/129140	0.03
	AD#078	0	c.2989C > T	p.Arg997Trp	Uncertain significance/amyotrophic lateral sclerosis.	1/113370	0.02
*FIG4*	AD#080	0	c.2200G > A	p.Glu734Lys	Uncertain significance/NR	15/113494	0.04
	AD#097	0	c.2467C > T	p.Gln823Ter*	Pathogenic/NR	7/129138	0.03
*FUS*	AD#039	0	c.430_447del	p.Gly144_Tyr149del	Conflicting interpretations of pathogenicity. Pathogenic (1); likely pathogenic (1); uncertain significance (1)/NR	10/113750	0.03
	AD#033	1.5	c.681_686del	p.Gly230_Gly231del	Conflicting interpretations of pathogenicity benign (1) uncertain significance (1)/NR	56/117710	
	AD#049	3.5					0.11
*MAPT*	AD#039	0	c.121G > A	p.Ala41Thr	Uncertain significance/Alzheimer disease?	6/128512	0.03
	AD#017	3.5	c.454G > A	p.Ala152Thr	Conflicting interpretation of pathogenicity uncertain significance (2) benign (1) likely benign (2)/neurodegeneration	297/129002	0.39
*NOTCH3*	AD#085	0	c.1505C > T	p.Ser502Phe	Uncertain significance/NR	9/75540	0.04
	AD#071	3	c.3315C > T	p.Gly1105=	NR/NR/prediction: likely benign	1/113330	0.02
	AD#099	0	c.3535A > G	p.Asn1179Asp	NR/NR/prediction: likely pathogenic	NR	NA
	AD#074	3.5	c.4461C > T	p.Gly1487=	NR/NR/prediction: likely benign	NR	NA
	AD#093	3.5	c.5816-6C > T		NR/NR prediction: likely benign	6/113474	0.03
*OPTN*	AD#010	3.5	c.448C > T	p.Leu150=	NR/NR prediction: likely benign	7/129170	0.03
	AD#091	3.5	c.941A > T	p.Gln314Leu	Conflicting interpretations of pathogenicity pathogenic (1) uncertain significance (1)/amyotrophic lateral sclerosis	38/129076	0.08
	AD#069	0	c.1401 + 4A > G		Uncertain significance/amyotrophic lateral sclerosis	17/129180	0.05
	AD#053	0	c.1643G > A	p.Arg548Gln	Uncertain significance/NR	9/129122	0.03
*SQSTM1*	AD#066	0	c.315C > T	p.Cys105=	NR/NR prediction: likely benign	NR	NA
	AD#092	3.5	c.960G > A	p.Gly320=	NR/NR prediction: likely benign	1/81718	0.02
	AD#032	3.5	c.1175C > T	p.Pro392Leu	Conflicting interpretations of pathogenicity pathogenic (4) likely pathogenic (1) uncertain significance (2) benign (1)/paget disease of bone	173/128718	0.27
*TYROBP*	AD#077	0	c.140T > C	p.Val47Ala	Uncertain significance – Alzheimer disease, early onset?	14/128556	0.04
*UBQLN2*	AD#095	0	c.1461C > A	p.Thr487=	Conflicting interpretations of pathogenicity uncertain significance (1); benign (5); likely benign (1)/NR	787/87391	1

Only variants not previously defined as benign/likely benign in ClinVar and MAF < 0.01 referred to the European (non-Finnish) population reported on GnomAD v2.1.1. were selected and reported. Prediction of variant pathogenicity were reported in the Supplementary Tables 2–4. AD, Alzheimer disease, FH, family history, NR. Not reported RE, reported. ^1^Population allele frequencies referred to the European (non-Finnish) population reported on GnomAD v2.1.1. ^2^*P*-value Fisher’s exact test. *Variant homozygous.

**TABLE 5:** Rare variants in AD-risk genes *ABAC7* and *SORL1* identified in this study.

Gene	ID patient	FH score	Nucleotide change	Protein change	Pathogenicity reported in ClinVar/HGMD	Possible role in EOAD	Frequency GnomAD (EU)^1^	*P*-value^2^
*ABCA7*	AD#009	0	c.2126_2132del AGCAGGG	p.Glu709Al afsTer86	Conflicting interpretations of pathogenicity; risk factor Uncertain significance (1) likely benign (1)/NR	Reported AD risk (De Roeck et al., 2019) risk factor	250/104264	0.18
	AD#052	3.5	c.2476G > A	p.Gly826Arg	NR/NR Prediction: likely pathogenic	Risk factor	95/126126	0.08
	AD#088	0	c.2629G > A	p.Ala877Thr	NR/NR Prediction: likely benign	Likely benign	1014/127604	0.41
	AD#006	0	c.3412A > C	p.Ser1138Arg	NR/NR Prediction: likely pathogenic	Risk factor	1/76428	0.01
	AD#009	0	c.3472 + 5G > C		NR/NR Prediction: likely benign	Possibly affecting splicing (Le Guennec et al., 2016) – Risk factor	2/112726	0.01
	AD#083	0	c.4343G > A	p.Gly1448Asp	NR/NR Prediction: likely benign	Likely benign	72/127544	0.07
	AD#007	0	c.4795G > A	p.Val1599Met	Likely benign/autism?	Likely benign	554/129150	0.28
	AD#028	3.5	c.5570 + 5G > C		Uncertain significance/Alzheimer disease?	Reported AD risk (De Roeck et al., 2019) risk factor	432/114436	0.05
	AD#099	0						
*SORL1*	AD#094	3.5	c.133G > T	p.Asp45Tyr	NR/NR Prediction: likely pathogenic	Risk factor	4/39936	0.02
	AD#052	3.5	c.1805C > T	p.Ser602Leu	NR/Alzheimer disease?	Risk factor	NR	NA
	AD#096	0	c.3346A > G	p.Ile1116Val	Benign/Alzheimer disease, late-onset?	Likely benign	1065/129098	0.41
	AD#062	0	c.4077C > T	p.Cys1359=	Uncertain significance/NR	Risk factor	33/129196	0.03
	AD#097	0	c.5448T > C	p.Tyr1816=	Benign/NR	Likely benign	285/128866	0.18
	AD#088	0	c.6150A > G	p.Glu2050=	NR/NR Prediction: likely benign	Likely benign	1/113030	0.01

AD, Alzheimer disease, FH, family history, NA, not applicable, NR. Not reported. ^1^Population allele frequencies referred to the European (non-Finnish) population reported on GnomAD v2.1.1, expressed as Allele count (Alt/total). ^2^*P*-value Fisher’s exact test, BH correction.

Among the VUS, two were identified in the *MAPT* gene. Although *MAPT* pathogenic variants are typically associated with FTD (Cruts et al., 2012), these variants have already been reported in patients with AD (Cochran et al., 2019). One of them (p.Ala152Thr) has been identified as a risk factor for several neurodegenerative diseases, including AD (Sydow et al., 2016). The other *MAPT* variant (p.Ala41Thr) occurred in a patient also carrying a pathogenic mutation in the *FUS* gene (AD#039). Due to the heterogeneity of genetic factors contributing to neurodegeneration, pathogenic/likely pathogenic variants and VUS have been considered as weak allele risk factors (Dilliott et al., 2021). Carriers of these variants were equally distributed between the familial and sporadic groups (Fisher exact test *p*-value = 1).

### Missing heritability: Role of rare variants in other Alzheimer’s disease-risk factor genes

**FIGURE 1:**
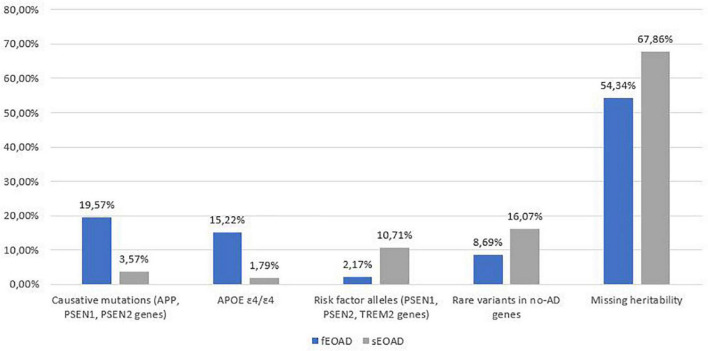
Summary of genetic variants identified in the 102 AD patients, divided into familial (fEOAD) and sporadic patients (sEOAD). Causative mutations included diagnostic and strong contributor alleles in *APP*, *PSEN1*, and *PSEN2* genes, moderate risk alleles included variant not strictly pathogenic in *APP*, *PSEN1*, *PSEN2*, and *TREM2* genes, rare variants in no-AD genes included pathogenic/likely pathogenic/VUS (uncertain significant variants or with major evidence of pathogenicity in ClinVar and/or HGMD) variants in genes causative for other type of dementia (Supplementary Table 1).

Rare pathogenic variants in AD causative genes, homozygosity for *APOE* ε4/ε4, risk factor alleles and likely pathogenic variants in genes related to other forms of neurodegenerative dementias explain 46% of familial and 32% of sporadic patients in our cohort (Figure 1). In recent years, more than 40 AD-associated genes/loci have been identified by GWAS, and subsequent sequencing projects have highlighted the role of rare variants in these genes in EOAD (Bellenguez et al., 2020; Wightman et al., 2021). In a pilot cohort of EOADs without causative variants (*n* = 30) we analyzed 34 genes (Supplementary Table 1), identified as susceptibility factors for AD, for the presence of rare and possibly pathogenic variants.

Most of the variants identified were in the *ABCA7* (*n* = 8; one in two patients) and *SORL1* (*n* = 6) genes (Table 5). Of these, two in the *ABCA7* gene were previously reported PTV (protein truncating variants) (Table 5). Among them, the c.2126_2132delAGCAGG p.Glu709AlafsTer86 variant was classified as a contributor to the disease (De Roeck et al., 2019). We also found three rare missense variants in the coding sequence of *SORL1*, one of which (p.Asp45Tyr) was classified as a likely pathogenic variant. Among the possibly pathogenic variants in *ABCA7* and *SORL1*, five had a positive association in our cohort compared to the frequency in the European population (Table 5). The remaining missense or silent variants were considered as rare benign variants (Table 5 and Supplementary Tables 2–4).

Additionally, 26 rare variants of potential interest (Table 6) were identified in 17 of the 32 other AD susceptibility genes analyzed (Supplementary Table 1). All identified variants were singleton except for the *FERMT2* p.Thr513Met variant. Only three variants were reported in ClinVar or HGMD databases, all classified as VUS. Variants in the *BIN1* gene (p.Asn232Lys, and c.1462-3C > T) were linked to myopathy, while the variant in *PTK2B* was associated with Parkinson’s disease. As for the remaining variants, eight were classified as likely pathogenic by *in silico* analyses (Table 6 and Supplementary Tables 2–4).

**TABLE 6:** Rare variants in GWAS genes identified in this study.

Gene	ID patient	FH score	Nucleotide change	Protein change	Pathogenicity ClinVar/HGMD	Frequency GnomAD (EU)^1^	*P*-value
*ADAM10*	AD#060	0	c.112A > G	p.Asn38Asp	NR/NR prediction: likely benign	NR	NA
	AD#065	3.5	c.556dupC	p.Gln186ProfsTer19	NR/NR prediction: likely pathogenic	NR	NA
*BIN1*	AD#095	0	c.696C > A	p.Asn232Lys	Uncertain significance (myopathy)/NR	98/129022	0.08
	AD#009	0	c.865G > A	p.Ala289Thr	NR/NR prediction: likely benign	3/94156	0.01
	AD#082	0	c.1462-3C > T		Uncertain significance (myopathy)/NR	16/128710	0.02
*CLU*	AD#096	0	c.509C > T	p.Thr170Met	NR/NR prediction: likely benign	2/113698	0.01
*CR1*	AD#062	0	c.4956G > A	p.Pro1652=	NR/NR prediction: likely benign	NR	NA
	AD#075	0	c.4356T > C	p.Cys1452=	NR/NR prediction: likely benign	1084/128002	0.50
*ELAVL1*	AD#010	3.5	c.765C > T	p.Ala255=	NR/NA prediction: likely benign	29/129192	0.04
*EP300*	AD#098	0	c.2194C > T	p.Pro732Ser	NR/NR prediction: likely pathogenic	NR	NA
*EPHA1*	AD#010	3.5	c.928A > G	p.Ile310Val	NR/NR prediction: likely benign	1/113284	0.01
*FERMT2*	AD#028	3.5	c.1077G > C	p.Gly359=	NR/NA prediction: likely benign	1083/127414	0.41
	AD#058	0	c.1538C > T	p.Thr513Met	NR/NA prediction: likely pathogenic	487/129124	0.05
	AD#060	0					
*INPP5D*	AD#090	0	c.470G > A	p.Arg157Gln	NR/NR prediction: likely pathogenic	300/127840	0.18
	AD#089	3.5	c.2085C > T	p.Pro695=	NR/NR prediction: likely benign	107/128314	0.08
*MARK2*	AD#090	0	c.1611C > T	p.Ser537=	NR/NA prediction: likely benign	5/108824	0.01
*MARK4*	AD#060	0	c.1553C > T	p.Pro518Leu	NR/NR prediction: likely benign	196/129058	0.14
*PICALM*	AD#014	0	c.1231G > C	p.Ala411Pro	NR/NR prediction: likely pathogenic	321/128802	0.18
*PLCG2*	AD#032	3.5	c.3379C > A	p.Pro1127Thr	NR/NR prediction: likely pathogenic	3/128682	0.01
	AD#083	0	c.408G > A	p.Ala136=	NR/NR prediction: likely benign	1/128708	0.01
*PTK2B*	AD#010	3.5	c.2591C > T	p.Ala864Val	NR/Parkinson disease?	275/129154	0.18
*RIN3*	AD#022	0	c.2377T > C	p.Tyr793His	NR/NA prediction: likely pathogenic	896/129136	0.40
*TOMM40*	AD#082	0	c.384C > G	p.Asn128Lys	NR/NR prediction: likely benign	4/113466	0.01
*ZCWPW1*	AD#052	3.5	c.1834C > T	p.Leu612=	NR/NR prediction: likely benign	1126/128540	0.41
	AD#097	0	c.314A > G	p.Glu105Gly	NR/NA prediction: likely pathogenic	623/128268	0.30
	AD#088	0	c.283-5T > G		NR/NR prediction: likely benign	1072/128080	0.41

Only variants not previously defined as benign/likely benign in ClinVar and MAF < 0.01 referred to the European (non-Finnish) population reported on GnomAD v2.1.1. were reported. Novel variants have been classified as “likely pathogenic” if at least three tools out of the four used showed potentially pathogenic effects (Supplementary Table 2). AD, Alzheimer disease, FH, family history; NR, not reported; NA, not applicable.

^1^Population allele frequencies referred to the European (non-Finnish) population reported on GnomAD v2.1.1, expressed as Allele count (Alt/total).

In conclusion, 63% of the examined patients presented at least one likely pathogenic variant or a rare VUS in AD-susceptibility genes (Tables 5, 6), suggesting that they might also confer risk for the development of EOAD. Familial and sporadic patients did not show a different distribution of these variants.

## Discussion

Clarifying the genetic and molecular basis of EOAD and its clinical variability is crucial for improving diagnostic screening and developing more effective, and possibly preventive, disease modifying treatments.

Since a concern in genetic studies of AD patients is that at least some variants are private or specific to certain populations, we analyzed a well-characterized single-center cohort of 102 patients by a NGS panel including AD causative genes, risk factors and genes involved in other types of dementia.

We found a pathogenic mutation in one of the three AD-causing genes in 8.82% of patients, in agreement with the expected maximum of 11% reported in the literature (Mendez, 2019). These include a novel, likely pathogenic variant, *PSEN1* c.253C > T p.Leu85Phe. According to Guerreiro algorithm (Guerreiro et al., 2010), this variant can be defined as probably pathogenic: it maps to the first transmembrane domain, is defined as damaging by *in silico* tools (Supplementary Table 2), a causative mutation in the same codon (p.Leu85Pro) has been described in patients with EOAD (Ataka et al., 2004), and the residue is conserved in *PSEN2* (p.Leu91). *PSEN2* mutations are considered very rare but have the highest frequency in Spain and Italy (Cai et al., 2015). In agreement, we identified four possibly pathogenic missense variants in this gene, two possibly pathogenic missense variants, p.Gly223Ala and p.Leu396Phe, both in patients with no family history of AD, and two rare variants, p.Met174Val and p.Arg71Trp, which have a questionable classification. These variants were initially described as possibly pathogenic (Guerreiro et al., 2010) but subsequently found in healthy controls. Thus, they are currently classified as non-pathogenic (Alzforum Mutation database^3^). We demonstrated a positive association with the risk of developing EOAD for the *PSEN2* p.Met174Val variant, compared with the non-Finnish European allele frequency reported in GnomAD, having found it in two patients with EOAD and positive family history. This variants could be considered a contributing factor to the disease.

Given the previously reported common mechanisms between AD and other dementias (Bartoletti-Stella et al., 2018; Giau et al., 2019), we also collected data on likely pathogenic rare variants in genes implicated in other dementias. Of the 33 rare variants identified in 12 genes, 10 were pathogenic or likely pathogenic, nine VUS, equally distributed between the familial and sporadic EOAD (Table 4). Most of them were in genes causal for the ALS/FTD continuum. Thus, our study supports the view that AD and other neurodegenerative diseases might represent shades of the same disease spectrum, and that extended genetic testing of causative genes for other degenerative dementias should be offered to patients diagnosed with EOAD ([Bibr B5][Bibr B25]). In agreement with this hypothesis, Spina et al., 2021, found at least one non-AD neuropathological diagnosis in 98% of patients with EOAD. Due to the heterogeneity of genetic factors contributing to neurodegeneration, and the equal distribution between sporadic and familial forms, we consider pathogenic/hypothetical pathogenic variants and variants with uncertain significance in these genes to be weak risk factors (Dilliott et al., 2021).

Concerning the main known risk factors for AD, homozygosity for *APOE* ε4 allele has previously been reported to be associated with a significantly increased risk of EOAD than of LOAD, regardless of family history (van Duijn et al., 1994; BLACKER et al., 1997; Cochran et al., 2019); in agreement, it reaches 7.84% in our dataset. This evidence confirms previous data suggesting that APOE may strongly influence AD risk at younger ages, but as age increases, the effect of *APOE* is reduced, and other risk variants start to play a more significant role in AD risk (Bellou et al., 2020). These include variants in *ABCA7*, *SORL1*, and *TREM2* gene. Regarding *TREM2*, a significant exome-wide association between the p.Arg47His variant and the risk of EOAD has been reported while rare *TREM2* variants have been associated with Aβ deposition, Aβ uptake by microglia, and increased tau in CSF (Gratuze et al., 2018). Together, these data suggest a connection with EOAD neuropathologic findings, and both the amyloid and tau hypotheses of AD (Ayodele et al., 2021). Furthermore, in patients with autosomal-dominant AD, CSF Aβ42 decline, cortical atrophy and cognitive impairment were lower in patients with high soluble receptor stub (Morenas-Rodríguez et al., 2022). In the *TREM2* coding region we found five rare VUS (one patient carried two variants), two of which (p.Arg136Gln, p.Thr223Ile) showed a higher allele frequency in EOAD than the European population reported on GnomAD (Table 3). The p.Arg136Gln variant causes the substitution of an arginine residue required for optimal binding of extracellular ligands, and other known missense variants were found in familial and early onset forms of the disease (Sirkis et al., 2016). In agreement with a previous study (Bellenguez et al., 2017), we considered these variants as risk factor/weak contributor to the disease.

Our results are therefore consistent with previous findings and strengthen the potential role of these rare variants as risk factors.

Recent meta-analyses and GWAS have shown a fivefold increased risk, similar to that of *APOE*-ε4 carriers, of developing EOAD with rare variants in *SORL1* (Ayodele et al., 2021) and a study that used the Exome Aggregation Consortium (ExAC; ExAC browser is not available anymore, and it is now part of the gnomAD) database established that pathogenic *SORL1* variants increase AD risk by 12-fold, as well as causing an earlier age of onset (58.6 ± 5.2 years) (Holstege et al., 2017). Moreover, protein-truncating variants in *SORL1* were observed exclusively in AD patients, and are highly penetrant, whereas two *SORL1* missense mutations (p.R1303C and p.G1732A) and a splice site variant (c.3050-2A > G) have been shown to segregate with disease in families affected by autosomal dominant AD (Holstege et al., 2017). Among the six rare variants in the *SORL1* gene identified in our cohort, p.Asp45Tyr and p.Ser402Leu were found in two familial patients. The variant p.Asp45Tyr was never previously reported and classified as possibly pathogenic.

In addition, we identified five unreported missense variants in *ABCA7*, (Table 2), a gene containing risk factors for developing both EOAD and LOAD due to its role in amyloid clearance and decreasing Aβ production through interference with APP processing (Satoh et al., 2015; De Roeck et al., 2017; Sleegers and Van Broeckhoven, 2019). In addition, *ABCA7* has been shown to provide a greater predisposition to develop AD than the APOE ε4 allele in African American adults. To date, four autosomal dominant AD families are known in which rare *ABCA7* PTC and missense variants segregated with the disease (Hoogmartens et al., 2021). Although our variants are missense, they may likely act as essential contributors to EOAD susceptibility, albeit with variable penetrance (Khani et al., 2022).

Lastly, in EOAD without causative mutations or *APOE* ε4/ε4, we analyzed 34 genes previously identified as susceptibility factors for AD. We found an average of 0.6 rare variants in each patient in 14 of them, also confirming in EOAD patients a high amount of risk factors.

Overall, the extended genetic analysis of a well-defined cohort of Italian EOAD patients showed that in cases without dominant pathogenic variants in *PSEN1*, *PSEN2* and *APP*, there is an enrichment for multiple pathogenic/likely pathogenic variants in genes associated with risk factors for AD, covering 38% of our cohort, supporting the extreme genetic heterogeneity of EOAD. Independently of family history, a high proportion of EOAD patients carried genetic risk factors, suggesting oligogenic determinism, particularly in individuals without familiarity. Therefore, our data also highlight the role of rare variants especially in sporadic EOAD, each exerting a moderate to high pathogenic effect and corroborate an extended analysis to identify variants implicated in the disease.

We also confirmed enrichment of *APOE* ε4/ε4 homozygosity and rare pathogenic/likely pathogenic variants in *SORL1*, *TREM2*, and *ABCA7* (Khani et al., 2022). Additionally, we found a substantial proportion of pathogenic variants in several autosomal dominant genes, causal in other dementias or previously identified as risk factors for AD, most often in patients with positive familiarity, providing evidence to the hypothesis that many different rare mutations usually detected only in a single family or in small populations could be causal in familial EOAD.

In summary, our study describes new pathogenic variants in AD linked genes, *PSEN1*, *ABCA7*, *SORL1*, and contributes to disentangle the broad genetic landscape of Italian EOAD. The results suggest that a systematic application of comprehensive genetic assessments could aid in the interpretation of early-onset dementia cases by providing a molecular basis to their phenotypic heterogeneity and potentially in enabling future personalized medicine approaches.

## Data availability statement

The datasets presented in this study can be found in online repositories and the study is deposited in the European Nucleotide Archive (ENA, https://www.ebi.ac.uk/ena/) EMBLEBI repository, accession number PRJEB55143 (ERP140024).

## Ethics statement

Ethical approval of the study was obtained by the ethical board Area Vasta Emilia Centro, (AVEC). The patients/participants provided their written informed consent to participate in this study.

## Author contributions

AB-S and MT: manuscript drafting and revising, data collection, analysis, and interpretation of data. GM, FA, LB, NM, MS-M, SB, SL, MS, RP, EF, PC, RL, and PP: data collection and revision of the manuscript. SC: study concept and design, drafting and revising the manuscript, analysis and interpretation of data, and study supervision. All authors contributed to the article and approved the submitted version.

## Footnotes

1
www.umd.be/HSF/

2
https://fathmm.biocompute.org.uk/fathmm-xf/

3
https://www.alzforum.org/mutations
