# Supplementary material for: Dementia-related genetic variants in an Italian population of early-onset Alzheimer’s disease
Source: Front Aging Neurosci. 2022 Sep 5;14:969817. doi: 10.3389/fnagi.2022.969817 (PMC9484406; doi:10.3389/fnagi.2022.969817)
Supplement: Supplementary file 2 [file Data_Sheet_1.docx]

Supplementary Material: Dementia-related genetic variants in an Italian population of Early-Onset Alzheimer’s Disease

# Supplementary Tables

**Table S1: Genes analyzed in this study.** Key: AD, Alzheimer disease, RefSeq, Reference sequence, REF, reference. *Genes analyzed in a subgroup of patients (n=30)

| **Symbol** | **Name** | **RefSeq** | **Role in AD [REF]** |
| --- | --- | --- | --- |
| *APP* | amyloid beta precursor protein | NM_000484.3 | AD causative gene [Cacace et al., 2016] |
| *PSEN1* | presenilin 1 | NM_000021.3 | AD causative gene [Cacace et al., 2016] |
| *PSEN2* | presenilin 2 | NM_000447.2 | AD causative gene [Cacace et al., 2016] |
| *C9orf72* | chromosome 9 open reading frame 72 | NM_018325.4 | Other dementia gene [Bartoletti-stella et al., 2018] |
| *CCNF* | Cyclin F | NM_001761.2 | Other dementia gene [Bartoletti-stella et al., 2018] |
| *CHCHD10* | coiled-coil-helix-coiled-coil-helix domain containing 10 | NM_213720.2 | Other dementia gene [Bartoletti-stella et al., 2018] |
| *CHMP2B* | charged multivesicular body protein 2B | NM_014043.3 | Other dementia gene [Bartoletti-stella et al., 2018] |
| *CSF1R* | Colony stimulating factor 1 receptor | NM_005211.2 | Other dementia gene [Bartoletti-stella et al., 2018] |
| *DCTN1* | Dynactin subunit 1 | NM_004082.4 | Other dementia gene [Bartoletti-stella et al., 2018] |
| *FIG4* | FIG4 phosphoinositide 5-phosphatase | NM_014845.5 | Other dementia gene [Bartoletti-stella et al., 2018] |
| *FUS* | FUS RNA binding protein | NM_004960.3 | Other dementia gene [Bartoletti-stella et al., 2018] |
| *GRN* | Granulin | NM_002087.3 | Other dementia gene [Bartoletti-stella et al., 2018] |
| *ITM2B* | integral membrane protein 2B | NM_021999.4 | Other dementia gene [Bartoletti-stella et al., 2018] |
| *MAPT* | Microtubule associated protein tau | NM_005910.5 | Other dementia gene [Bartoletti-stella et al., 2018] |
| *NOTCH3* | notch 3 | NM_000435.2 | Other dementia gene [Bartoletti-stella et al., 2018] |
| *OPTN* | Optineurin | NM_021980.4 | Other dementia gene [Bartoletti-stella et al., 2018] |
| *SQSTM1* | sesquestosome 1 | NM_003900.4 | Other dementia gene [Bartoletti-stella et al., 2018] |
| *TARDBP* | TAR DNA binding protein | NM_007375.3 | Other dementia gene [Bartoletti-stella et al., 2018] |
| *TBK1* | TANK binding kinase 1 | NM_013254.3 | Other dementia gene [Bartoletti-stella et al., 2018] |
| *TYROBP* | TYRO protein tyrosine kinase binding protein | NM_003332.3 | Other dementia gene [Bartoletti-stella et al., 2018] |
| *UBQLN2* | Ubiquilin 2 | NM_013444.3 | Other dementia gene [Bartoletti-stella et al., 2018] |
| *VCP* | Valosin-containing protein | NM_007126.4 | Other dementia gene [Bartoletti-stella et al., 2018] |
| *ABCA7** | ATP binding cassette subfamily A member 7 | NM_019112.4 | AD risk gene [Dourlen et al., 2019] |
| *ABI3** | ABI family member 3 | NM_016428.3 | AD risk gene [Conway et al., 2018] |
| *ADAM10** | ADAM metallopeptidase domain 10 | NM_001110.2 | AD risk gene [Dourlen et al., 2019] |
| *AKT1** | AKT serine/threonine kinase 1 | NM_005163.2 | AD risk gene [Liu et al., 2015] |
| *APOE* | apolipoprotein E | NM_000041.2 | AD risk gene [Dourlen et al., 2019] |
| *BIN1** | Bridging integrator 1 | NM_139343.1 | AD risk gene [Dourlen et al., 2019] |
| *CASS4** | Cas scaffold protein family member 4 | NM_020356.4 | AD risk gene [Dourlen et al., 2019] |
| *CD2AP** | CD2 associated protein | NM_012120.2 | AD risk gene [Dourlen et al., 2019] |
| *CD33** | CD33 molecule | NM_001772.3 | AD risk gene [Dourlen et al., 2019] |
| *CELF1** | CUGBP Elav-Like Family Member 1 | NM_001025596.3 | AD risk gene [Dourlen et al., 2019] |
| *CLU** | Clusterin | NM_001831.2 | AD risk gene [Dourlen et al., 2019] |
| *CR1** | Complement C3b/C4b receptor 1 (Knops blood group) | NM_000573.3 | AD risk gene [Dourlen et al., 2019] |
| *ELAVL1** | ELAV like RNA binding protein 1 | NM_001419.3 | AD risk gene [Raj et al., 2018] |
| *EP300** | E1A binding protein p300 | NM_001429.4 | AD risk gene [Xie et al., 2020] |
| *EPHA1** | [EPH receptor A1](https://www.ncbi.nlm.nih.gov/gene/2041) | NM_005232.5 | AD risk gene [Wang et al., 2015] |
| *EPHA4** | EPH receptor A4 | NM_004438.5 | AD risk gene [Simón et al., 2009] |
| *FERMT2** | Fermitin family member 2 | NM_006832.3 | AD risk gene [Dourlen et al., 2019] |
| *HTRA2** | HtrA serine peptidase 2 | NM_013247.5 | AD risk gene [Westerlund et al., 2011] |
| *INPP5D** | Inositol polyphosphate-5-phosphatase D | NM_001017915.2 | AD risk gene [Dourlen et al., 2019] |
| *MARK2** | Microtubule affinity regulating kinase 2 | NM_001039469.2 | AD risk gene [Gu et al., 2013] |
| *MARK4** | Microtubule affinity regulating kinase 4 | NM_001199867.2 | AD risk gene [Pathak et al., 2020] |
| *MEF2C** | Myocyte enhancer factor 2C | NM_002397.5 | AD risk gene [Tang et al., 2016] |
| *MS4A4A** | Membrane spanning 4-domains A4A | NM_024021.4 | AD risk gene [Dourlen et al., 2019] |
| *MS4A6E** | Membrane spanning 4-domains A6E | NM_139249.3 | AD risk gene [Ma et al., 2016] |
| *NME8** | NME/NM23 family member 8 | NM_016616.5 | AD risk gene [Liu et al., 2014] |
| *PICALM** | Phosphatidylinositol binding clathrin assembly protein | NM_007166.4 | AD risk gene [Dourlen et al., 2019] |
| *PLCG2** | Phospholipase C gamma 2 | NM_002661.5 | AD risk gene [Dourlen et al., 2019] |
| *PTK2B** | Protein tyrosine kinase 2 beta | NM_004103.4 | AD risk gene [Dourlen et al., 2019] |
| *RIN3** | Ras and Rab interactor 3 | NM_024832 | AD risk gene [Dourlen et al., 2019] |
| *SLC24A4** | Solute carrier family 24 member 4 | NM_153646.4 | AD risk gene [Dourlen et al., 2019] |
| *SORL1** | Sortilin related receptor 1 | NM_003105.6 | AD risk gene [Dourlen et al., 2019] |
| *TOMM40** | Translocase of outer mitochondrial membrane 40 | NM_006114.3 | AD risk gene [Dourlen et al., 2019] |
| *TP53INP1** | tumor protein p53 inducible nuclear protein 1 | NM_033285.4 | AD risk gene [Escott-Price et al., 2014] |
| *TREM2* | triggering receptor expressed on myeloid cells 2 | NM_003105.5 | AD risk gene [Dourlen et al., 2019] |
| *TRIP4** | Thyroid hormone receptor interactor 4 | NM_016213.5 | AD risk gene [Ruiz et al., 2014] |
| *ZCWPW1** | zinc finger CW-type and PWWP domain containing 1 | NM_017984.6 | AD risk gene [Dourlen et al., 2019] |

**Table S2**: Pathogenic prediction of missense variants by *in-silico* tools

| **Gene** | **Variant** | **Polyphen2** | **M-CAP** | **CADD score** | **Mutation Taster** | **Final classification based on in silico prediction^1^** |
| --- | --- | --- | --- | --- | --- | --- |
| *ABAC7* | c.2476G>A p.Gly826Arg | Possibly Damaging | Possibly pathogenic | 20.7 | Disease causing | Likely pathogenic |
| *ABCA7* | c.2629G>A p.Ala877Thr | Benign | NA | 15.67 | Polymorphism | Likely benign |
| *ABCA7* | c.3412A>C p.Ser1138Arg | Possibly Damaging | Possibly pathogenic | 26.4 | Disease causing | Likely pathogenic |
| *ABCA7* | c.4343G>A p.Gly1448Asp | Possibly Damaging | Possibly pathogenic | 13.21 | Polymorphism | Likely benign |
| *ADAM10* | c.112A>G p.Asn38Asp | Benign | Likely benign | 17.07 | Disease Causing | Likely benign |
| *BIN1* | c.865G>A p.Ala289Thr | Benign | Likely benign | 14.96 | Polymorphism | Likely benign |
| *CCNF* | c.353T>C p.Val118Ala | Possibly damaging | Likely benign | 22.7 | Disease causing | Likely pathogenic |
| *CCNF* | c.656T>C p.Leu219Pro | Possibly damaging | Possibly pathogenic | 16.44 | Disease causing | Likely pathogenic |
| *CLU* | c.509C>T p.Thr170Met | Probably damaging | Likely Benign | 16.45 | Polymorphism | Likely benign |
| *CSF1R* | c.1400C>T p.Thr467Met | Possibly damaging | Possibly pathogenic | 9.584 | Polymorphism | Likely benign |
| *CSF1R* | C.1477A>G p.Ser493Gly | Benign | Likely benign | 23.3 | Polymorphism | Likely benign |
| *CSF1R* | c.2850C>A p.His950Gln | Benign | Likely benign | 0.110 | Polymorphism | Likely benign |
| *CSF1R* | c.2851C>A p.Leu951Met | Probably damaging | Likely benign | 17.07 | Polymorphism | Likely benign |
| *DCTN1* | c.1361T>C p.Val454Ala | Benign | Possibly pathogenic | 23.1 | Disease causing | Likely pathogenic |
| *DCTN1* | c.1555A>G p.Lys519Glu | Probably damaging | Possibly pathogenic | 29.1 | Disease causing | Likely pathogenic |
| *EP300* | c.2194C>T p.Pro732Ser | Probably damaging | Possibly pathogenic | 25.1 | Disease Causing | Likely pathogenic |
| *EPHA1* | c.928A>G p.Ile310Val | Benign | Possibly pathogenic | 0.02 | Polymorphism | Likely benign |
| *FERMT2* | c.1538C>T p.Thr513Met | Possibly damaging | Likely benign | 24.2 | Disease Causing | Likely pathogenic |
| *INPP5D* | c.470G>A p.Arg157Gln | Possibly damaging | Possibly pathogenic | 21.4 | Polymorphism | Likely pathogenic |
| *MARK4* | c.1553C>T p.Pro518Leu | Benign | Likely benign | 21.1 | Polymorphism | Likely benign |
| *NOTCH3* | c.3535A>G p.Asn1179Asp | Probably damaging | Possibly pathogenic | 24.2 | Disease Causing | Likely pathogenic |
| *PICALM* | c.1231G>C p.Ala411Pro | Possibly damaging | Likely benign | 20.1 | Disease causing | Likely pathogenic |
| *PLCG2* | c.3379C>A p.Pro1127Thr | Probably damaging | Possibly pathogenic | 26.8 | Disease causing | Likely pathogenic |
| *PSEN1* | c.253C>T p.Leu85Phe | Probably Damaging | Probably Pathogenic | 26.6 | Disease causing | Likely pathogenic |
| *RIN3* | c.2377T>C p.Tyr793His | Probably damaging | Possibly pathogenic | 26.8 | Disease causing | Likely pathogenic |
| *SORL1* | c.133G>T p.Asp45Tyr | Benign | Possibly Pathogenic | 23 | Disease causing | Likely pathogenic |
| *TOMM40* | c.384C>G p.Asn128Lys | Benign | Likely benign | 23.6 | Disease causing | Likely benign |
| *ZCWPW1* | c.314A>G p.Glu105Gly | Probably damaging | Possibly pathogenic | 32 | Polymorphism | Likely pathogenic |

^1^ Variants have been classified as “likely pathogenic” if at least three tools out of the four used showed potentially pathogenic effects.

**Table S3**: Pathogenic prediction of silent variants by *in-silico* tools

| **Gene** | **Variant** | **CADD score** | **Mutation Taster** | **FATHMM-XF** | **Final classification based on in silico prediction^1^** |
| --- | --- | --- | --- | --- | --- |
| *CR1* | c.4956G>A p.Pro1652= | 1.585 | Polymorphism | Benign | Likely benign |
| *CR1* | c.4356T>C p.Cys1452= | 2.464 | Polymorphism | Benign | Likely benign |
| *ELAVL1* | c.765C>T p.Ala255= | 12.21 | Disease Causing | Benign | Likely benign |
| *FERMT2* | c.1077G>C p.Gly359= | 10.58 | Disease Causing | Benign | Likely benign |
| *INPP5D* | c.2085C>T p.Pro695= | 0.789 | Disease Causing | Benign | Likely benign |
| *MARK2* | c.1611C>T p.Ser537= | 11.01 | Disease Causing | Benign | Likely benign |
| *NOTCH3* | c.3315C>T p.Gly1105= | 13.01 | Disease Causing | Benign | Likely benign |
| *NOTCH3* | c.4461C>T p.Gly1487= | 12.02 | Disease Causing | Benign | Likely benign |
| *OPTN* | c.448C>T p.Leu150= | 5.598 | Disease Causing | Benign | Likely benign |
| *PLCG2* | c.408G>A p.Ala136= | 5.410 | Disease Causing | Benign | Likely benign |
| *SORL1* | c.6150A>G p.Glu2050= | 10.06 | Disease Causing | Benign | Likely benign |
| *SQSTM1* | c.315C>T p.Cys105= | 12.34 | Disease Causing | Benign | Likely benign |
| *SQSTM1* | c.960G>A p.Gly320= | 0.096 | Polymorphism | Benign | Likely benign |
| *ZCWPW1* | c.1834C>T p. Leu612= | 8.682 | Disease Causing | Benign | Likely benign |

^1^ Variants have been classified as “likely pathogenic” if at least two tools out of the three used showed potentially pathogenic effects.

**Table S4**: Pathogenic prediction of splicing variants by *in-silico* tools. Key: MUT, mutation. NR, not reported. WT, wild type.

| **Gene** | **Variant** | **Human Splicing finder** | **NNsplice** | **MaxEnt** | **Final classification^1^** |
| --- | --- | --- | --- | --- | --- |
| *ABAC7* | c.3472+5G>C | No significant impact on splicing signals | WT 0.60 / MUT 0.46 | WT 7.45/MUT 7.16 | Likely benign |
| *NOTCH3* | c.5816-6C>T | No significant impact on splicing signals | WT 0.91/MUT 0.94 | No significant impact on splicing signals. | Likely benign |
| *ZCWPW1* | c.283-5T>G | No significant impact on splicing signals | WT 0.99/MUT 0.94 | WT 11.96/MUT 9.72 | Likely benign |

^1^ Variants have been classified as “likely Pathogenic” if at least two tools out of the three used showed potentially pathogenic effects.

Figure S1: Dimensionality reduction plots performed with Principal Component Analysis (PCA, left panel) and t-distributed stochastic neighbor embedding (t-SNE, Jaccard similarity used as metric, right panel) of the overall genetic profile of samples in the biggest technical batch. The plots show an overall homogenous genetic background in our EOAD cohort, with no confounders caused by the geographical origin of the patients.


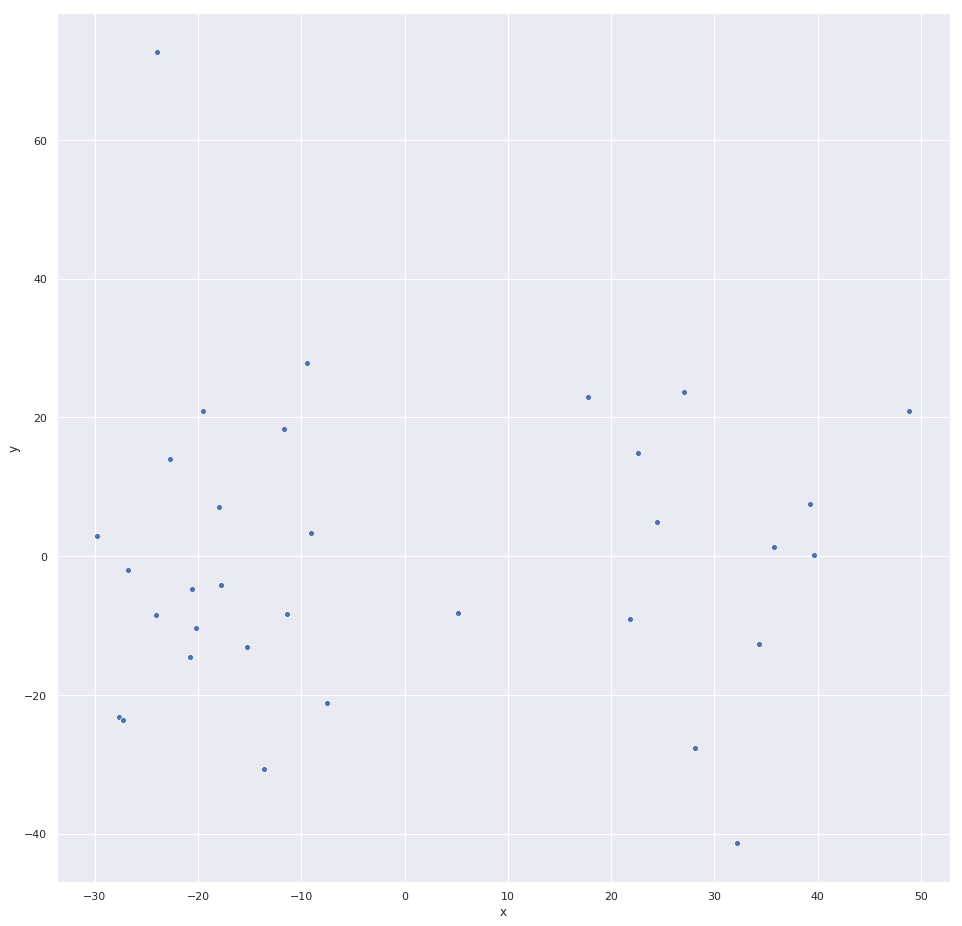

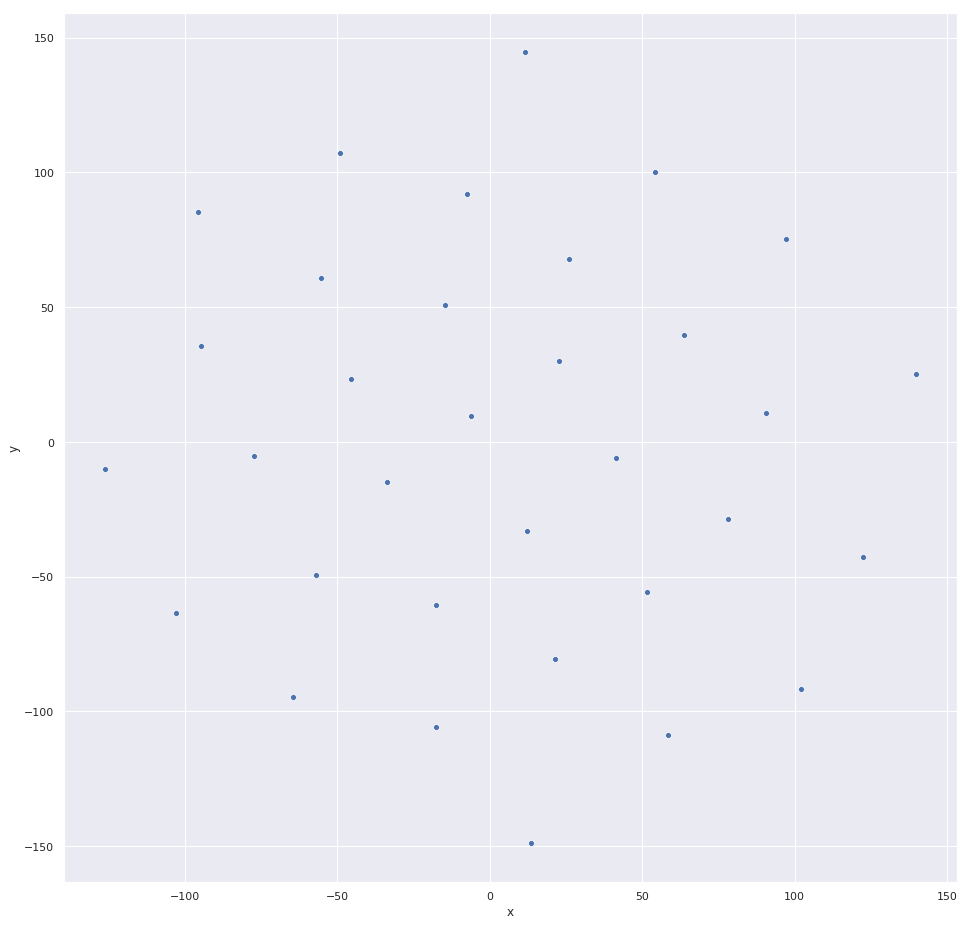


# Supplementary References

Conway OJ, Carrasquillo MM, Wang X, et al. ABI3 and PLCG2 missense variants as risk factors for neurodegenerative diseases in Caucasians and African Americans. Mol Neurodegener. 2018;13(1):53. Published 2018 Oct 11. doi:10.1186/s13024-018-0289-x

Escott-Price V, Bellenguez C, Wang LS, et al. Gene-wide analysis detects two new susceptibility genes for Alzheimer's disease. PLoS One. 2014;9(6):e94661. Published 2014 Jun 12. doi:10.1371/journal.pone.0094661

Gu GJ, Wu D, Lund H, et al. Elevated MARK2-dependent phosphorylation of Tau in Alzheimer's disease. J Alzheimers Dis. 2013;33(3):699-713. doi:10.3233/JAD-2012-121357

Liu SY, Zhao HD, Wang JL, et al. Association between Polymorphisms of the AKT1 Gene Promoter and Risk of the Alzheimer's Disease in a Chinese Han Population with Type 2 Diabetes. CNS Neurosci Ther. 2015;21(8):619-625. doi:10.1111/cns.12430

Liu Y, Yu JT, Wang HF, et al. Association between NME8 locus polymorphism and cognitive decline, cerebrospinal fluid and neuroimaging biomarkers in Alzheimer's disease. PLoS One. 2014;9(12):e114777. Published 2014 Dec 8. doi:10.1371/journal.pone.0114777

Ma J, Zhang W, Tan L, et al. MS4A6A genotypes are associated with the atrophy rates of Alzheimer's disease related brain structures. Oncotarget. 2016;7(37):58779-58788. doi:10.18632/oncotarget.9563

Pathak GA, Zhou Z, Silzer TK, Barber RC, Phillips NR; Alzheimer's Disease Neuroimaging Initiative, Breast and Prostate Cancer Cohort Consortium, and Alzheimer's Disease Genetics Consortium. Two-stage Bayesian GWAS of 9576 individuals identifies SNP regions that are targeted by miRNAs inversely expressed in Alzheimer's and cancer. Alzheimers Dement. 2020 Jan;16(1):162-177. doi: 10.1002/alz.12003. PMID: 31914222.

Raj T, Li YI, Wong G, et al. Integrative transcriptome analyses of the aging brain implicate altered splicing in Alzheimer's disease susceptibility. Nat Genet. 2018;50(11):1584-1592. doi:10.1038/s41588-018-0238-1

Roses AD, Lutz MW, Amrine-Madsen H, et al. A TOMM40 variable-length polymorphism predicts the age of late-onset Alzheimer's disease. Pharmacogenomics J. 2010;10(5):375-384. doi:10.1038/tpj.2009.69

Ruiz A, Heilmann S, Becker T, et al. Follow-up of loci from the International Genomics of Alzheimer's Disease Project identifies TRIP4 as a novel susceptibility gene. Transl Psychiatry. 2014;4(2):e358. Published 2014 Feb 4. doi:10.1038/tp.2014.2

Simón AM, de Maturana RL, Ricobaraza A, Escribano L, Schiapparelli L, Cuadrado-Tejedor M et al. Early changes in hippocampal Eph receptors precede the onset of memory decline in mouse models of Alzheimer’s disease. J Alzheimers Dis 2009; 17: 773–786.

Tang SS, Wang HF, Zhang W, et al. MEF2C rs190982 polymorphism with late-onset Alzheimer's disease in Han Chinese: A replication study and meta-analyses. Oncotarget. 2016;7(26):39136-39142. doi:10.18632/oncotarget.9819

Wang HF, Tan L, Hao XK, et al. Effect of EPHA1 genetic variation on cerebrospinal fluid and neuroimaging biomarkers in healthy, mild cognitive impairment and Alzheimer's disease cohorts. J Alzheimers Dis. 2015;44(1):115-123. doi:10.3233/JAD-141488

Westerlund M, Behbahani H, Gellhaar S, et al. Altered enzymatic activity and allele frequency of OMI/HTRA2 in Alzheimer's disease. FASEB J. 2011;25(4):1345-1352. doi:10.1096/fj.10-163402

Xie L, Varathan P, Nho K, Saykin AJ, Salama P, Yan J. Identification of functionally connected multi-omic biomarkers for Alzheimer's disease using modularity-constrained
